# Priority knowledge gaps for schistosomiasis research and development in the World Health Organization Africa Region

**DOI:** 10.1186/s40249-025-01285-w

**Published:** 2025-03-17

**Authors:** Pauline N. Mwinzi, Moses Chimbari, Khadime Sylla, Maurice R. Odiere, Nicholas Midzi, Eugene Ruberanziza, Sylvian Mupoyi, Humphrey D. Mazigo, Jean T. Coulibaly, Uwem Friday Ekpo, Moussa Sacko, Sammy M. Njenga, Louis-Albert Tchuem-Tchuente, Anouk N. Gouvras, David Rollinson, Amadou Garba, Elizabeth A. Juma

**Affiliations:** 1 Expanded Special Project for Elimination of NTDs, WHO Regional Office for Africa, Brazzaville, Republic of Congo; 2 University of KwaZulu-Natal, College of Health Sciences, Durban, South Africa; 3 Universite Cheikh Anta Diop, Dakar, Senegal; 4 Centre for Global Health Research, Kenya Medical Research Institute, Kisumu, Kenya; 5 African Research Network for Neglected Tropical Diseases, KCCR, KNUST, Kumasi, Ghana; 6 National Institute of Health Research, Ministry of Health and Childcare, Harare, Zimbabwe; 7 The END Fund, New York City, NY USA; 8 Department of Tropical Medicine, University of Kinshasa, Kinshasa, Democratic Republic of the Congo; 9 National Programme for the Fight Against Bilharzia and Intestinal Parasitoses, Kinshasa, Democratic Republic of Congo; 10 Department of Medical Parasitology and Entomology, School of Medicine, Catholic University of Health and Allied Sciences, Mwanza, Tanzania; 11 Faculty of Biosciences, Félix Houphouët-Boigny University, Abidjan, Côte d’Ivoire; 12 Swiss Center for Scientific Research in Côte d’Ivoire, Abidjan, Côte d’Ivoire; 13 Department of Pure and Applied Zoology, Federal University of Agriculture Abeokuta, Abeokuta, Nigeria; 14 Department of Zoology, Akwa Ibom State University, Ikot Akpaden, Akwa Ibom State Nigeria; 15 Department of Diagnostic and Biomedical Research, National Institute of Public Health Research, Bamako, Mali; 16 Eastern and Southern Africa Centre of International Parasite Control, Kenya Medical Research Institute, Nairobi, Kenya; 17 Centre for Schistosomiasis and Parasitology, Yaoundé, Cameroon; 18 Laboratory of Parasitology and Ecology, Faculty of Sciences, University of Yaoundé I, Yaoundé, Cameroon; 19 National Programme for the Control of Schistosomiasis and Intestinal Helminthiasis, Ministry of Public Health, Yaoundé, Cameroon; 20 Global Schistosomiasis Alliance, London, UK; 21 Global NTD Programme, World Health Organization, Geneva, Switzerland

**Keywords:** Schistosomiasis, Neglected tropical diseases, Preventive chemotherapy, One Health approach, Morbidity indicators, Diagnostic tools, Mass Drug Administration, Zoonotic transmission, Water, sanitation and hygiene, Intermediate snail hosts

## Abstract

Schistosomiasis, also known as bilharzia, is a widespread neglected tropical disease (NTD) in Africa, with more significant research and development (R&D) challenges and gaps compared to other preventive chemotherapy NTDs (PC-NTDs) like onchocerciasis, lymphatic filariasis, and trachoma. In response to this challenge, some global initiatives have advocated for bridging this gap, focusing on coordinated engagement with research donors. In this opinion article we highlight key R&D priorities for combating schistosomiasis in the WHO Africa region. These include defining morbidity indicators, expanding prevention, and developing innovative diagnostics, treatments, and public health strategies like test-and-treat. We emphasize integrating efforts with broader health campaigns, assessing zoonotic transmission through One Health, and using environmental surveillance tools like xenomonitoring and eDNA. We stress the need to study climate and environmental impacts on transmission, zoonotic transmission, schistosome hybridization, and snail ecology, advancing snail control, and developing vaccines, while calling for new treatments beyond praziquantel, addressing drug resistance, and improving access for children and remote populations. Further, operational research should refine hotspot interventions, enhance water, sanitation and hygiene integration, and address socio-cultural barriers. Lastly, sustainable funding and global collaboration are vital to achieve 2030 NTD Roadmap goals.

**Graphical Abstract:**

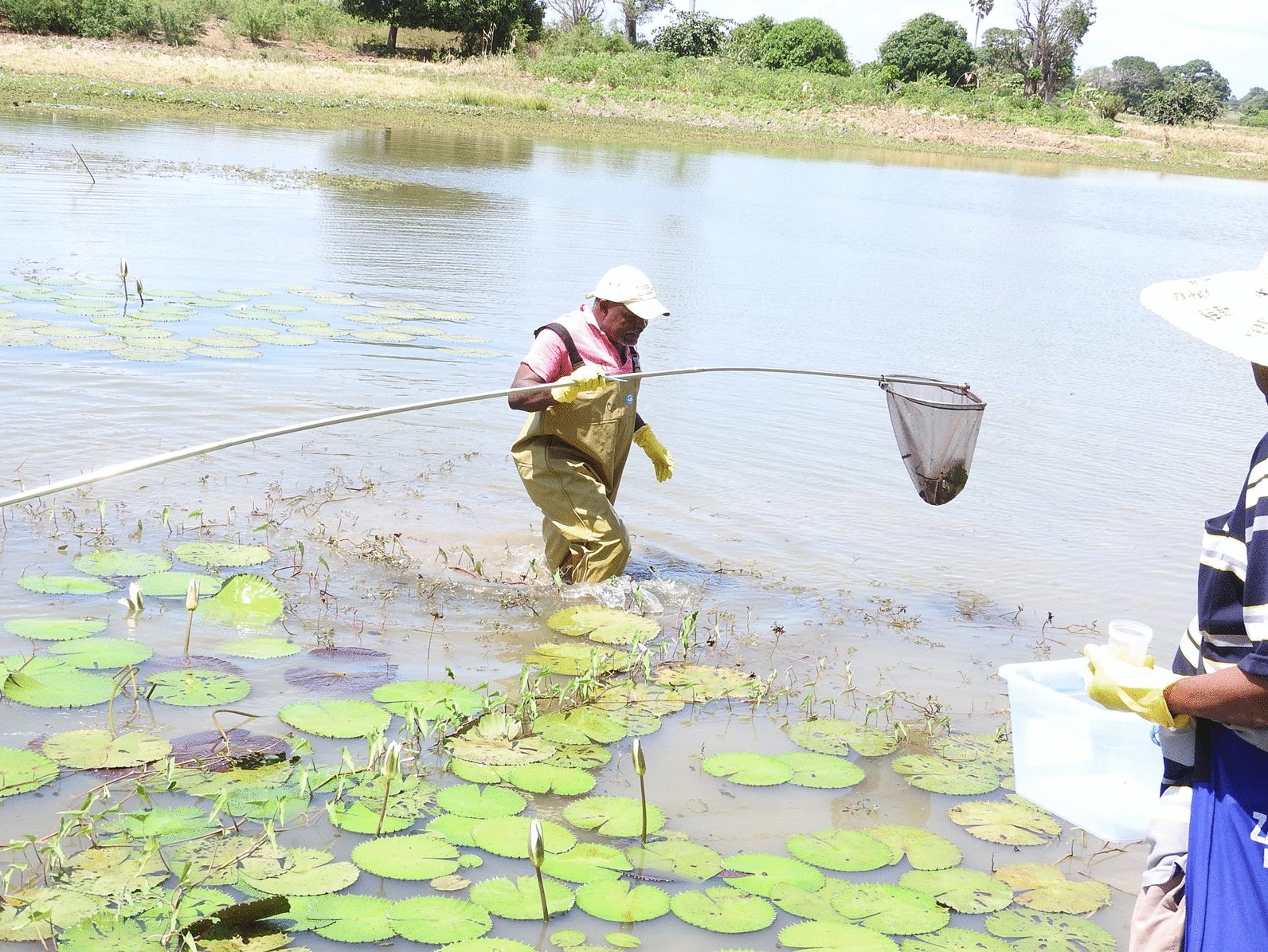

## Background

Schistosomiasis also known as bilharzia, caused by parasitic trematodes of the genus *Schistosoma* is one of the most widespread neglected tropical diseases (NTDs) in the African region, but also significantly one of those lagging behind in terms research and development (R&D), compared to other NTDs prioritized under the Expanded Special Project for Elimination of Neglected Tropical Diseases (ESPEN), including onchocerciasis, lymphatic filariasis, and trachoma [1]. Various global collaborative initiatives have been established in the past decade to propel R&D for NTDs including schistosomiasis. These include the formation of the ESPEN https://espen.afro.who.int/, the NTD NGO Network (NNN)—https://www.ntd-ngonetwork.org/ to coordinate the work of organizations engaged in the fight against NTDs, and other NTD-related alliances; and the establishment of the Coalition for Operational Research on NTDs https://www.cor-ntd.org/ as a leading scientific body focused on NTDs which is contributing to funding research in resource limited settings. In the Africa region the African Research Network for Neglected Tropical Diseases (ARNTD) https://arntd.org/ has been building capacity among young and mid-career researchers. Notably, it is the formation of the Global Schistosomiasis Alliance (GSA) https://www.eliminateschisto.org/ which has helped bring a specific focus to schistosomiasis thus closing the gap between it and other NTDs in terms of R&D.

Building on previous advocacy on schistosomiasis control and research gaps [2–5], The NTD Roadmap 2030 identifies several research gaps and needs for schistosomiasis, emphasizing the importance of comprehensive strategies to meet 2030 targets [6]. Key actions include defining morbidity indicators, extending preventive chemotherapy (PC) to all population groups in need, implementing targeted snail control, and developing new diagnostic tests and interventions, including alternatives to praziquantel [6]. The roadmap highlights the need for improved scientific understanding, particularly regarding transmission pathways, specific snail species, zoonotic reservoirs, and the spectrum of morbidities associated with the disease. It calls for developing standardized, sensitive diagnostics for various settings and uses, including surveillance and clinical use, and creating a biorepository for diagnostic development. Additionally, the roadmap suggests operational research to refine current strategies, including micro-targeting of interventions, exploring new medicines, and promoting WASH and behavioural change interventions. The Roadmap 2030 also underscores the importance of cross-sectoral governance and sufficient resource allocation, including domestic financing and health care capacity strengthening for R&D in this area. Addressing these knowledge gaps requires global solidarity and coordinated efforts among researchers, communities, public health professionals, governments, funders and international organizations. Increased funding, enhanced surveillance, and innovative research are essential to fill these gaps and move closer to the control and eventual elimination of schistosomiasis in Africa.

Responding to the NTD roadmap call [6], there is currently increased research on schistosomiasis in the WHO Africa Region. At the end of 2023, published research work from the Africa region on preventive chemotherapy [7–11] focused on efficacy of praziquantel [the drug of choice for schistosomiasis[7], evaluation of within-country schistosomiasis strategies [8], strategies to improve treatment compliance in the context of the new WHO schistosomiasis guidelines 2022 [9–11], and challenges and perspectives on implementing the new schistosomiasis guidelines [12, 13]. Much research focused on diagnostics [14–18], finding new serologic methods [15–17], alternative diagnostics to Kato-Katz and urine microscopy [18, 19], use of deep learning systems and artificial intelligence to enhance diagnostic tools [20–22] and diagnostics for female genital schistosomiasis (FGS) [17]. Reported mapping surveys [23–25] focused on precision mapping for community-level data and identification of transmission in new areas [24–26]. Much research was conducted on schistosomiasis morbidity including FGS [27–40], with most of the work focusing on diagnostics and prevalence surveys for FGS [27–36], integration of FGS into health systems [34–37], co-morbidities [38–40] and awareness of FGS among health care professionals [39, 40] and patients [41]. Deeper appreciation of the complexity of schistosomiasis transmission at the nexus of humans, animals and environment contributed to increased research on the One Health approach for schistosomiasis in 2023 [41]. There was continued research into intermediate snail hosts [42, 43]. With a new paediatric formulation at the verge of unveiling by the Paediatric Praziquantel Consortium, research into reaching paediatric populations [44] will provide valuable data towards implementation of treatments in this population. This opinion piece synthesizes recent evidence to identify and highlight the critical R&D gaps that must be addressed to advance schistosomiasis control and elimination efforts in the WHO African Region, to inform future strategies and prioritize areas for action to accelerate progress toward the 2030 targets.

## Main text

### Methodological approach

**Fig. 1:**
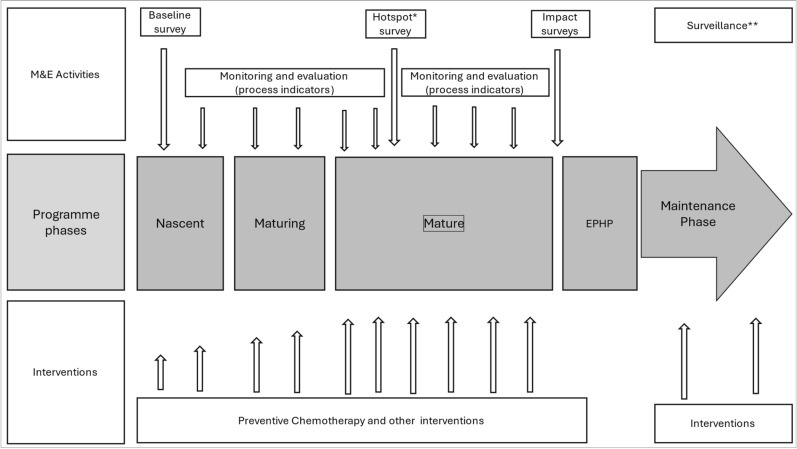
Phases of schistosomiasis control and elimination programmes. *To be conducted in selected areas (with suspected high transmission of schistosomiasis infection) after at least 2 years of PC, however, there is need to clearly define hotspots currently defined as “an area that demonstrates a < 1/3 reduction in prevalence of *Schistosoma* spp. infection between an initial survey (with prevalence ≥ 10%) and a follow up survey conducted after at least 2 years of preventive chemotherapy with effective (≥ 75%) treatment coverage” [42]. **After a country has been validated for EPHP, post-validation surveillance is recommended. However, surveillance is also recommended in any implementation unit that has reduced the frequency of PC distribution or achieved the EPHP target. *EPHP* elimination as public health problem, *PC* preventive chemotherapy

Leading schistosomiasis researchers in the WHO Africa Region reviewed the progress made by national schistosomiasis programmes in the World Health Organization Africa Region, as presented in the ESPEN portal, based on data shared by the national programmes with WHO, and published publicly on the portal (https://espen.afro.who.int). While some actions and tools are required throughout the control and elimination programme, different phases of the control and elimination programme require specific actions and tools. Thus, knowledge gaps were identified based on the programme phase of WHO AFRO countries as presented in the WHO schistosomiasis and soil-transmitted helminthiasis (STH) monitoring and evaluation (M&E) framework [45, 46] (Fig. 1). We classified the countries (Table 1) according to the programme phases outlined in Fig. 1. This progress was based on implementation of preventive chemotherapy as follows: nascent programmes as those with less than 3 annual PC rounds; maturing programmes as those that have conducted between 3–5 annual PC rounds with sub-optimal coverage; and mature programmes as those that have consistently conducted more than 5 annual PC rounds, with or without impact assessment. For the next categories, countries under elimination as public health problem (EPHP) are those that have attained elimination of schistosomiasis as a public health problem at national level as defined in the WHO NTD roadmap 2021–2030 (currently defined as < 1% proportion of heavy intensity infections), while those in sustaining EPHP phase are countries that have been validated for EPHP status by the WHO. Elimination of transmission is achieved when transmission of infections is fully interrupted in humans and other zoonotic reservoirs, defined as “Zero autochthonous incidence in humans for at least 5 consecutive years” and is followed by the post elimination surveillance phase. Despite ongoing control efforts and R&D progress, schistosomiasis remains a significant public health issue in many parts of Africa. We then considered significant knowledge gaps in each programme phase (Table 2) and in cross-cutting areas (Table 3).

**Table 1:** Programmatic phases for countries in the WHO Africa Region. *EPHP* elimination of schistosomiasis as a public health problem

Nascent programmes	Maturing programmes	Mature programmes	EPHP*	Sustaining EPHP*	Elimination of transmission	Post elimination surveillance
Equatorial Guinea Namibia South Africa	Angola Botswana Central African Republic Chad Congo Gabon Guinea-Bissau Nigeria South Sudan Zambia	Benin Burkina Faso Burundi Cameroon Côte d'Ivoire Democratic Republic of the Congo Eritrea Eswatini Ethiopia Gambia Ghana Guinea Kenya Liberia Madagascar Malawi Mali Mauritania Mozambique Niger Rwanda Sao Tome and Principe Senegal Sierra Leone Togo Uganda United Republic of Tanzania Zimbabwe	Algeria		Mauritius

**Table 2:** Overview of knowledge gaps in the Africa region categorized by control programme status. *WASH* water, sanitation, and hygiene

Programme phase	Research area	Identified gap	Proposed research focus	Target population	References
Nascent Programmes	Disease mapping and innovative approaches to fill data gaps	Inadequate data on transmission sites and precise prevalence data	Conduct mapping and prevalence studies using innovative and cost-effective approaches	Endemic regions	[6]
	Diagnostics	Reliance on labor-intensive, less sensitive diagnostics and lack of standardized, rapid tools	Develop and deploy rapid, sensitive, and specific point-of-care diagnostic tools	Populations in nascent programs	[46]
	Integration, monitoring and evaluation	Poor understanding of co-endemicity and absence of baseline datasets to inform decision-making	Study co-endemicity, establish baseline datasets, and integrate diagnostics into monitoring frameworks	National and endemic communities	
Maturing Programmes	Scaling up mass drug administration and Treatment Optimization	Irregular or incomplete mass drug administration (MDA) implementation, Challenges in achieving consistent MDA	Operational research to scale up MDA, optimize treatment strategies, identify persistent hotspots and develop tailored plans	Communities in endemic regions	[6, 46]
Fully Scaled Mature Programmes	Persistent hotspots/persistent transmission, and non-responding areas	Strategies for addressing persistent hotspots and areas with low treatment response, insufficient knowledge on factors influencing transmission including among others high baseline prevalence, potential drug resistance, low treatment coverage, environmental conditions, abundance of snail hosts, and human behaviors.	Develop strategies to address non-responding areas, including hotspot mapping and tailored interventions. Investigate environmental, genetic, and behavioral factors driving persistent transmission	Persistent hotspot communities/areas	[47–52]
	Integration with Other Health programs	Need for more research on integrating schistosomiasis control with other NTD control programs and broader public health interventions.	Research strategies for integrating and mainstreaming schistosomiasis control with NTD and other public health programs and routine health services	National health systems	[46]
	Hard-to-reach populations	Low treatment coverage in hard-to-reach areas in remote or marginalized populations, limited strategies for access and ensuring high treatment coverage.	Conduct social science studies to identify barriers and design strategies for reaching marginalized groups	Populations in hard-to-reach areas	[47–56]
	Morbidity management, morbidity indicators, residual morbidity.	Limited understanding of chronic schistosomiasis' long-term health impacts highlights the need for measurable morbidity indicators, comprehensive surveys, reliable markers, and innovative management strategies.	Conduct morbidity surveys, identify markers, and innovate management strategies for chronic schistosomiasis	Adults, Women of reproductive aga and children in endemic regions	[47–62]
	Drug efficacy and resistance	Concerns about potential drug resistance, insufficient research on addressing resistance, and inadequate continuous monitoring of drug efficacy.	Monitor praziquantel efficacy, research in new therapeutic agents, and improve drug accessibility	All populations undergoing MDA	[48]
	New therapeutic agents and access strategies	Need for research on new treatments, strategies to increase therapeutic coverage, and improving access to pediatric formulations of praziquantel.	Develop and test new therapeutic agents, pediatric formulations, and optimize therapeutic coverage, understanding optimal delivery platforms for pediatric praziquantel	All populations undergoing MDA, pre-school age children	[48]
	WASH Infrastructure	Lack of adequate water, sanitation, and hygiene (WASH) infrastructures and access, weak partnerships/linkages with WASH players	Study the role of WASH access and infrastructure in disease control, and develop integrated intervention models	Communities with poor WASH infrastructure	[57, 58]
	Environmental and behavioral factors	Persistent transmission due to environmental factors, and human behavior.	Research in environmental factors, and human behavior influencing transmission	Communities with inadequate access to safe water	[49–58]
	Co-infections	Inadequate understanding of the impact of schistosome co-infections with malaria, HIV/AIDS, TB and other infections	Study co-infection dynamics and their influence on clinical progression, disease severity, immune response, and treatment effectiveness	Endemic regions with overlapping diseases	[47–54]
Achieving and Sustaining EPHP	Water resource development (infrastructure), integration and coverage assessment	Insufficient data on WASH coverage, infrastructure, and its role in sustaining elimination, as well as limited understanding of the impact of water resource projects and socio-cultural factors on disease transmission and outcomes.	Evaluate WASH coverage and effectiveness, promote water resource development, and research culturally sensitive interventions and their integration into schistosomiasis elimination programs, including impacts on snail ecology and transmission patterns.	Communities in elimination phase affected by water development	[57, 58]
	Climate change: impact of environmental changes	Inadequate data on how climate change, water resource development, and land use changes impact schistosomiasis transmission and snail ecology.	Research the impact of climate change and water development projects on schistosomiasis transmission patterns	Communities in climate-sensitive areas	[6]
	Impact assessments after high coverage MDA	Lack of updated baseline data after over five years of high MDA coverage necessitates impact assessments to guide treatment adjustments.	Conduct impact assessments to set new baselines and inform adjustments to treatment strategies	Communities with several rounds of sustained high (≥75%) MDA coverage	[46]
	Treatment strategies and passive surveillance	Limited evidence on optimizing test-and-treat strategies for long-sustaining EPHP and passive surveillance	Research on test and treat protocols and their scale up, research on innovative passive surveillance and integration with routine healthcare services including reporting of indicators in the DHIS reporting tools.	All populations at risk	[6]
	Human behavior, cultural practices, culturally sensitive interventions, socioeconomic factors and gender sensitive approaches	Limited understanding of how human behavior, cultural practices, socioeconomic factors, and gender dynamics influence schistosomiasis exposure and outcomes, hindering the design of culturally appropriate and effective intervention strategies.	Research the influence of cultural norms and behaviors on disease dynamics to develop context-specific, culturally, and gender-sensitive strategies for effective behavior change and intervention design.	Women and vulnerable groups in endemic areas	[6]
	Snail ecology and snail control in persistent Hotspots	Limited understanding of snail vectors and their ecology. Inadequacy of current snail contol measure hinders effective hotspot control, emphasizing the need for innovative, eco-friendly, and sustainable solutions to reduce transmission and environmental impacts	Exploring snail ecology and hybridization is essential to inform targeted control measures, with a focus on developing eco-friendly and effective strategies for persistent hotspots.	Communities in persistent hotspots near water bodies	[59, 62]
	Hybridization and drug efficacy	Need for studies on hybridization of schistosome species and the efficacy of praziquantel on these hybrids. Limited understanding of hybrid schistosome species and their response to praziquantel	Study parasite hybridization and evaluate drug efficacy	Endemic regions with hybrid species	[6]
	Surveillance methodologies	Need for improved surveillance systems to monitor the emergence or reemergence of schistosomiasis in previously cleared areas. Inadequate systems to monitor snail populations and disease re-emergence	Develop integrated surveillance methodologies, including the use of eDNA to detect and monitor snail habitats	Health and surveillance teams	[46]
	Zoonotic transmission	Gaps in understanding zoonotic transmission and identifying interventions to address zoonotic reservoirs. Poor understanding of zoonotic reservoirs and their role in sustaining transmission	Investigate zoonotic reservoirs and design interventions to mitigate zoonotic transmission	Communities near zoonotic reservoirs	[6]
	Vaccine development	Lack of an effective vaccine for schistosomiasis. Need for identifying viable vaccine targets and developing effective vaccines for humans and animals	Identifying viable vaccine targets and developing effective vaccines for both humans and animals	Endemic populations and livestock	[6]

**Table 3:** Overview of knowledge gaps in the Africa region categorized by cross-cutting areas. *WASH* water, sanitation, and hygiene, *FGS* female genital schistosomiasis; *NTD* neglected tropical diseases

Research area	Identified gap	Proposed research focus	Target population	References
Preventive chemotherapy	Emerging drug resistance	Research on resistance mechanisms and development of alternative medicines to complement praziquantel, build models to simulate surveillance systems and optimize outbreak detection and response strategies	Populations undergoing repeated MDA	[6, 85, 86]
	Limited drug options	Accelerate the development of new anti-schistosomal drugs and explore combination therapies	All populations in endemic areas	[6]
	Reinfection after treatment, drug resistance	Research strategies to reduce reinfection by targeting infection reservoirs through integrated approaches. Assess the risk of drug resistance and the long-term efficacy of current treatments through predictive modeling	Treated individuals in endemic regions	[6, 85, 86]
	Dependency on drug donations	Evaluate the sustainability of donation programs and explore self-reliant distribution and access models; innovative domestic resource mobilization strategies	Communities relying on donated medicines	
Diagnostics	Low sensitivity and specificity of current diagnostics, especially limitation of Kato-Katz for light intensity Infections	Develop innovative and standardized point-of-care diagnostic tools with high sensitivity and specificity based on current TPP. Research alternative diagnostic methods such as biomarkers, glycoproteins, and parasite DNA detection in bodily fluids	Endemic populations	[6, 46]
	Need for biomarkers for non-invasive testing	Conduct biomarker studies to identify secretome markers in blood and urine for non-invasive and sensitive diagnostics	Vulnerable populations requiring frequent testing	[6]
	Inaccessibility of adult schistosomes for direct infection estimation	Develop surrogate measurement techniques and improve standardization for estimating infection intensity	All schistosomiasis-affected populations	[6]
	Biological variations in egg excretion	Study factors such as diurnal variation and tissue fibrosis affecting egg excretion to refine diagnostic tools	Endemic regions with diverse populations	
Schistosomiasis morbidity	Morbidity indicators	Develop measurable indicators to assess the morbidity caused by schistosomiasis as per the NTD Roadmap	Global schistosomiasis programs	[6]
	Lack of gold standard diagnostics for FGS	Develop accurate and scalable diagnostic tools, including PCR and antigen detection methods	Women in endemic regions	[33, 68, 69]
	Efficacy of praziquantel for FGS	Research optimal timing and frequency of praziquantel treatment to improve outcomes for FGS	Women affected by FGS	
	Integration of FGS into reproductive health services	Develop integrated care approaches combining parasitological treatment with routine reproductive health and menstrual hygiene services	Women and healthcare providers	[71, 72]
	FGS co-morbidity with HIV, cervical cancer, bladder cancer and viral susceptibility	Evaluate the association between FGS and increased risk of HIV to develop targeted interventions, Investigate the link between *S. haematobium* infections and bladder cancer, as well as susceptibility to viral infections	Women in HIV and FGS co-endemic regions	[73]
	Psychosocial and stigma aspects	Assess the mental health impact and stigma associated with FGS and design community awareness initiatives	Affected individuals and communities	[6]
	Economic and health impact of FGS	Assess the economic burden and health impacts of FGS to inform policies and resource allocation	Policymakers and affected communities	[6]
	Male genital schistosomiasis (MGS)	Investigate health impacts, treatment efficacy, and diagnostic methods for MGS	Men in endemic regions	[74]
	Multisectoral multidisciplinary collaboration	Foster transdisciplinary research to integrate insights from epidemiology, veterinary science, and environmental science	Researchers and policymakers	[6]
One Health	Zoonotic transmission	Investigate zoonotic reservoirs and their role in maintaining schistosomiasis transmission, develop models to capture complex transmission patterns and heterogeneity of infection	Communities near wildlife and livestock	[6]
	Interaction of human activities with schistosomiasis	Study the impact of activities like agriculture and dam construction on disease prevalence	Populations in areas with major infrastructure projects	[6]
	Integrated surveillance systems	Develop and implement integrated systems to monitor infection in human, animal, and snail populations	National and regional health programs	
	Wildlife reservoirs	Assess the role of wildlife in the disease cycle and its implications for control measures	Regions with high wildlife density	
	Limited knowledge of snail vectors' ecological needs and the effects of altered water conditions on their parasite-hosting capacity hampers effective ecological control.	Investigate how ecological and habitat changes impact snail populations and transmission dynamics, focusing on the effects of water conditions on vector susceptibility and capacity	Regions with changing water conditions	[81, 84]
	Schistosome species diversity and hybridization	Investigate the impact of hybridization between human and animal schistosomes on adaptation, evolution, and transmission	Endemic populations and zoonotic reservoirs	[62, 76–79]
Climate Change Impact on schistosomiasis	Climate change impact: Heat stress effects on snail infection and schistosomiasis transmission are unclear, while rising temperatures expand transmission and disrupt control.	Research should examine how climate change affects transmission dynamics, snail habitats, and *Schistosoma* adaptation, using predictive models and heat stress studies to guide adaptive strategies and address emerging risks.	Communities in climate-sensitive regions and previously non-endemic regions	[82, 84–86]
	Emerging transmission patterns due to environmental changes	Modeling and surveillance systems to monitor climate-driven changes in snail habitats and schistosomiasis transmission	Communities in climate-sensitive areas	[86]
	Lack of precise climate models to predict environmental changes affecting snail habitats and Schistosoma lifecycle	Develop localized climate models to assess environmental changes and their impacts on transmission dynamics	Communities in climate-sensitive regions	[82, 84]
	Limited understanding of human and socioeconomic influences on climate-driven disease transmission	Assess how human behaviors and socioeconomic shifts interact with climate change to affect schistosomiasis patterns	Vulnerable communities	[82, 84]
	Need for integrated surveillance systems to monitor climate-driven changes and predict outbreaks	Implement surveillance systems incorporating technologies like environmental DNA (eDNA) for early detection	Health and monitoring systems	[82, 84]
	Lack of interdisciplinary approaches to evaluate and adapt control measures in response to evolving climate conditions	Combine insights from climatology, ecology, and public health to develop effective intervention and adaptation strategies	Endemic and high-risk populations	[82, 84]
Treating paediatric populations	Introduction of Paediatric Praziquantel (arPZQ)	Evaluation of implementation platforms and tailored approaches for the introduction of arPZQ in endemic regions	Young children <5yrs old in schistosomiasis-endemic areas	
	Long-term impact on children's development	Study the long-term effects of schistosomiasis and its treatment on growth, cognitive development, and future fertility	Children treated for schistosomiasis	
	Interaction with childhood conditions and malnutrition	Investigate the interaction between schistosomiasis, malnutrition, and other common childhood illnesses	Malnourished and co-morbid children	
	Accessibility of paediatric formulations	Evaluate barriers to accessing child-friendly drug formulations and design strategies to improve reach	Children in rural and underserved areas	
Snail intermediate host research gaps	Need for improved snail control technologies	Develop cheaper, effective, and environmentally friendly snail control methods, including plant-based molluscicides	Communities near schistosomiasis-prone water bodies	[6, 46]
	Snail host genetic diversity	Use molecular techniques like DNA barcoding to study genetic diversity within snail genera (e.g., *Bulinus* and *Biomphalaria*)	Snail populations in endemic regions	
	Compatibility between snails and schistosomes	Explore the physiological mechanisms of compatibility between specific schistosome and snail species	Snail-schistosome interaction hotspots	
	Biological control of snails	Revisit biological control methods while considering predator-prey dynamics and promoting sustainable alternatives to niclosamide	Communities relying on water bodies	
	Snail immune systems	Study mechanisms by which snail immune systems combat schistosome infections	Endemic regions with high infection rates	[82–84]
	Molluscan pheromones and chemoattractants	Investigate how miracidia locate snail hosts using pheromones/chemoattractants for potential control strategies	Snail populations in endemic regions	
	Emerging technologies (eDNA and microbiome manipulation)	Use eDNA for identifying waterbodies with intermediate snail species and explore microbiome manipulation for schistosome control	Waterbodies with endemic snail populations	
WASH	Limited access to safe water and sanitation facilities	Evaluate water treatment technologies, sanitation improvements, and access to safe water, Model the effects of WASH and socio-economic factors interventions on reducing schistosomiasis prevalence and transmission,	Communities relying on natural water bodies	[6, 85, 86]
	Integration of WASH with Behavioral Interventions	Develop integrated WASH and behavioral change strategies to reduce disease transmission	Endemic populations	
	Context-specific WASH solutions	Design and implement context-specific and sustainable WASH interventions, including safe washing facilities	Communities in endemic areas	
	Effectiveness of behavior change trategies	Assess the long-term effectiveness of behavior change strategies for promoting sustained healthy behaviors	Vulnerable populations	
	Monitoring and cost-effectiveness of WASH interventions	Develop indicators to monitor WASH effectiveness and evaluate the cost-effectiveness of interventions	National health programs	
	Barriers and cultural factors influencing hygiene practices	Identify cultural, motivational, and policy-related barriers to adopting effective hygiene and sanitation practices	Rural and underserved communities	
	Environmental and ecological dynamics	Investigate the role of environmental changes and snail ecology in disease transmission	Populations in high-transmission regions	
	Governance structures supporting WASH and behavior interventions	Explore effective governance and policy frameworks for implementing sustainable WASH and behavioral change programs	Policymakers and local authorities	

### Nascent programmes

Key knowledge gaps during this phase are the need for identifying potential transmission sites, disease distribution and prevalence data, and co-endemicity with other diseases for which integration of interventions is feasible. Precise, up-to-date data on the distribution and prevalence of schistosomiasis are lacking in many areas in the WHO Africa Region. This gap hinders effective targeting of control measures and resource allocation, especially in nascent programmes such as those of South Africa and Equatorial Guinea, the two countries in the WHO Africa Region currently classified in this category. Innovative approaches towards filling disease prevalence gaps are needed to enable progress. While development and deployment of rapid, sensitive, and specific diagnostic tools for both acute and chronic schistosomiasis are needed at all phases of the programme, having these in place for starting programmes sets an appreciable baseline dataset for appropriate policy decisions on timely treatment, and accurate assessment of control programmes in the continued monitoring and evaluation framework.

### Maturing programmes

For countries falling in this category, MDA interventions are started but not up to scale or are irregular. These countries include Angola, Botswana, Central African Republic, Chad, Congo, Gabon, Guinea-Bissau, Nigeria, South Sudan, and Zambia. While the countries are at different levels of maturing their programmes, operational research is needed in these countries to inform scale up and to optimize treatment coverage.

### Mature programmes

Neglected tropical diseases programmes in these countries have fully scaled up mass drug administration (MDA) to all endemic areas. Reaching populations in hard-to-reach areas is a priority, as well as ensuring high treatment coverage, and managing morbidity in adults. This involves extending preventive chemotherapy to all populations in need, ensuring access to essential NTD medicines, and implementing targeted snail control with updated guidelines. It also includes the continuation of micro-mapping and targeting to identify and treat affected populations accurately. Programmatic knowledge gaps in this phase include morbidity surveys, identifying morbidity markers and innovating for morbidity management; as well as social science studies on reaching hard to reach populations and understanding reasons for low coverage and existence of persistent hotspots (Fig. 1). There is a need for more research on the integration of schistosomiasis control with other NTD control programs and broader public health interventions to enhance efficiency and outcomes as elimination programmes mature, to facilitate their mainstreaming into routing health services. Furthermore, drug efficacy and resistance become a major concern with widespread annual MDAs. Continued monitoring of the efficacy of praziquantel, the primary drug used for schistosomiasis treatment, is necessary, particularly in the face of potential drug resistance. There is a need for research on new therapeutic agents and treatment strategies including strategies to increase therapeutic coverage to all population groups in need, and access to new paediatric formulations of praziquantel. In addition, once the primary objective of scaling up treatments to all in need is reached, residual disease morbidity and management becomes a priority**.** Greater understanding of the long-term health impacts of chronic schistosomiasis, including its contribution to anaemia, malnutrition, subfertility, cancer and cognitive impairment, and establishing a clear and measurable indicator to effectively assess the morbidity caused by schistosomiasis, is needed, including the gap in knowledge regarding the most effective strategies for managing and reducing morbidity. The impact of co-infections such as between schistosomiasis and malaria, HIV/AIDS, and helminth infections, are not well understood, even though such co-infections can influence disease severity, immune responses, and the effectiveness of treatment.

Further research is needed to come up with strategies for non-responding areas and hotspots. There is a need to understand contributing factors such as; high baseline prevalence and/or intensity [47], possible reduction in drug effectiveness [48], low treatment coverage (< 75%), environmental factors [49], high abundance of intermediate snail hosts [50, 51], low participation in MDA [52], reservoir hosts [53, 54], human behaviour [55, 56], inadequate water, sanitation, and hygiene [WASH] infrastructures and access [57, 58], and other intrinsic drivers such as human [59] and parasite genetics [60, 61].

### Elimination as a Public Health Problem Phase and sustaining EPHP status

Programmes that have conducted more than five years of high coverage annual MDA (> 75% community coverage) should conduct impact assessments to set a new baseline and consider adjusting treatment strategies if needed. Better understanding and deeper insights into how human behaviour, cultural practices, and how socioeconomic factors influence exposure to infection and disease outcomes is crucial for designing culturally sensitive and effective intervention strategies. Assessment of WASH coverage and its promotion as well as water resource development is critical during this phase.

### Elimination of transmission and post-elimination surveillance

To achieve elimination of transmission, snail control in persistent hotspots and high transmission areas as well as elimination of zoonotic schistosomiasis is required. In this regard it is important to study hybridization and efficacy of praziquantel on such hybrids. Schistosomiasis transmission is intricately linked to specific freshwater snails that serve as intermediate hosts for the parasites. There is a need for more detailed understanding of snail ecology, including factors that influence snail distribution, population dynamics, and interactions with the schistosome parasites to inform improved, targeted control approaches. During this phase the effects of environmental changes, such as climate change, water resource development [e.g., dams, irrigation], and land use changes, on schistosomiasis transmission should be monitored and their effects mitigated. These changes can alter habitats and the ecology of snail populations, potentially affecting disease transmission patterns, and research into these factors is needed as is the development of surveillance methodologies to monitor for the emergence and/or reemergence of schistosomiasis in previously clear areas. There is a need to develop and launch safer, cheaper, and effective snail control technology, mitigating environmental impacts. Furthermore, understanding zoonotic transmission and interventions to address zoonotic reservoirs can help achieve elimination of transmission. Another area that requires attention at this phase is vaccine development for both humans and animals to prevent reinfection and sustain elimination of transmission [62]. Despite significant research efforts over the decades including promising candidate vaccines such as rSh28GST (aka Bilhvax), Sm‐TSP‐2 (*S. mansoni* tetraspanin 2), Sm‐p80 (*S. mansoni* calpain), and Sm14 [63, 64], and radiation‐attenuated [RA] cercariae vaccine [65, 66], there is currently no vaccine available for schistosomiasis. Mathematical modelling shows that even a partly protective vaccine would play a role in reducing schistosome infections and hinder transmission [67]. Continued research to identify viable vaccine targets and develop effective vaccines is a critical knowledge gap.

### Cross-cutting scientific advances and research gaps

#### Preventing chemotherapy

The development of chemotherapy for schistosomiasis has faced significant challenges. Early treatments like arsenic-containing salvarsan and antimony potassium tartrate were highly toxic, and while praziquantel, introduced in 1982, is effective against adult schistosomes, it does not target younger forms and raises concerns about resistance with repeated mass treatments. The slow development of new drugs and lack of combination therapies limit options. Preventive chemotherapy coverage remains inadequate due to reinfections from untreated reservoirs and reliance on drug donations, which pose sustainability challenges. Urgent research is needed to address possible emergence of resistance, improve surveillance, and develop alternative medicines. Efforts should include evaluating preventive chemotherapy for all populations, refining access and distribution models, implementing targeted snail control, and addressing.

#### Schistosomiasis diagnostics

Schistosomiasis diagnostics face significant challenges due to inadequate tools with low sensitivity and specificity. The WHO NTD roadmap highlights the need for standardized point-of-care diagnostics. Diagnosing *S. haematobium* is hindered by the lack of a suitable animal model to correlate egg excretion with worm burden, while *S. mansoni* and *S. japonicum* diagnostics struggle with detecting light infections using Kato-Katz. Promising advancements include detecting glycoproteins and parasite DNA, but biomarker studies are needed for better targets. General challenges include reliance on surrogate measures, outdated calibration data, and biological factors affecting egg excretion. Improved, sensitive, and practical diagnostic methods are essential for better disease detection and control.

#### Urogenital schistosomiasis and morbidity

Significant progress has been made in understanding and managing urogenital schistosomiasis, particularly FGS, caused mainly by *S. haematobium.* FGS is characterized by egg deposition in the female genital tract, leading to symptoms like lesions, pain, bleeding, increased HIV risk, subfertility, and poor birth outcomes. Diagnosis lacks a "gold standard" and relies on identifying eggs in biopsies, with advances in polymerase chain reaction (PCR) testing and antigen detection showing promise [32, 68–70]. Praziquantel remains the primary treatment, with research ongoing into its efficacy, timing, and frequency specifically for FGS. Integrated care approaches combining treatment with reproductive health services and menstrual hygiene initiatives are critical [70–72]. Key research areas include raising awareness, developing scalable diagnostic and management guidelines, and understanding treatment effects on disease progression [73, 74]. The psychosocial aspects, including stigma and mental health impacts, require attention, alongside evaluations of FGS's association with HIV and its economic and health implications [74]. Male genital schistosomiasis (MGS) and its health impact also demand further study [75].

#### Schistosomiasis pathology

Research on schistosomiasis pathology faces significant gaps. Key areas include understanding historical disease impact, shifting transmission dynamics due to factors like population growth and irrigation, and the immunological mechanisms triggered by schistosome eggs in host tissues. More studies are needed on severe pathology in S. mansoni infections, genetic influences, and repeated exposures, as well as the pathogenesis of S. haematobium, including its role in bladder cancer, genital schistosomiasis, and viral susceptibility. Low-level infections and their role in transmission dynamics require attention. The urgency of eradication is heightened by the disease's severe health impacts, such as fibrosis hindering detection. The NTD Roadmap highlights the need for measurable indicators to assess schistosomiasis morbidity effectively.

#### One Health approach

The One Health approach, integrating human, animal, and environmental health, can advance schistosomiasis control by improving understanding of ecological factors affecting snail populations and environmental management practices like habitat modification and sanitation. Integrated surveillance systems for humans and snails are needed for more effective interventions. However, critical gaps remain in understanding zoonotic transmission, wildlife reservoirs, and the impact of human activities like agriculture and dam construction on disease prevalence. Climate change's influence on transmission dynamics also requires further study. Multidisciplinary collaboration across epidemiology, veterinary science, environmental science, and public health is essential to address these gaps and strengthen One Health strategies for schistosomiasis control.

#### Treating paediatric populations

Recent advancements in paediatric schistosomiasis treatment include the development of a child-friendly formulation of praziquantel (arpraziquantel, arPZQ), addressing issues with the original large, bitter tablets [75]. This breakthrough, led by the Pediatric Praziquantel Consortium, offers smaller, palatable tablets for easier ingestion. Despite approval by the European Medicines Agency, research is needed to implement arPZQ in endemic regions effectively. Additionally, gaps remain in understanding schistosomiasis's long-term effects on children's growth, cognitive development, fertility, and interactions with malnutrition and other childhood conditions, which may complicate recovery.

#### Snail intermediate host

Research on schistosome species and their snail hosts highlights key gaps. The WHO schistosomiasis guidelines 2022 [48] support environmentally friendly snail control, yet improved, cost-effective technologies are needed. Africa hosts five human-infecting species, including *S. mansoni, S. haematobium, S. rodhaini, S. intercalatum, S. guineensis* [61]. Molecular studies reveal hybridization between human and animal schistosomes, influencing adaptation and evolution, with notable cases in East and West Africa [76–80]. Hybrids between *S. haematobium* and *S. bovis* in Corsica raise concerns about wildlife reservoirs. DNA barcoding has enhanced snail classification, but compatibility mechanisms between schistosomes and snails remain unclear. Progress in using plant-based molluscicides, like *Phytolacca dodecandra*, offers promise [80]. Further, technologies like eDNA for detecting snail habitats and manipulating snail microbiomes should be explored. Understanding snail immune responses and miracidia-host interactions also remains critical [81–84].

## Operational research to improve effective WASH and behaviour interventions for prevention

Many African countries affected by schistosomiasis are still far from meeting WASH targets. Despite infrastructure improvements, some community members will continue to use natural water sources. Focal snail control, along with health education and behaviour change, can help ensure safe water use with a clear understanding of the risks. Endemic areas may need safe washing facilities at key water contact points. Operational research is essential to guide WASH and behaviour change interventions, ensuring they are effective, sustainable, and tailored to local contexts. This includes examining water treatment, sanitation, and safe water access, as well as testing community-led sanitation initiatives. Research should evaluate the cost-effectiveness of these interventions, the role of community engagement, and environmental factors in disease transmission. Additionally, studies should focus on the governance structures that support effective WASH, ensuring integration with broader health programs and achieving long-term disease prevention.

### Impact of climate change on schistosomiasis

Addressing the impact of climate change on schistosomiasis requires a multifaceted research approach. This includes developing localized climate models to predict environmental changes affecting snail habitats and the Schistosoma lifecycle. Research must investigate snail vector ecology, including how water condition changes influence parasite hosting. For example, while laboratory studies show heat pulses can make resistant *Biomphalaria glabrata* susceptible to *S. mansoni* [81], recent findings reveal minimal heat shock effects on infection prevalence in *B. glabrata* and *B. sudanica* and no impact on infection intensity [84]. Even small effects, however, can significantly influence transmission dynamics and public health [79]. Rising temperatures may enable transmission in new areas beyond current control programs, threatening the 2021–2030 Roadmap goals. Research should examine Schistosoma adaptation and range expansion while assessing human behaviour and socioeconomic shifts on transmission. Integrated surveillance, including environmental DNA (eDNA) technology, is critical for monitoring and predicting outbreaks. An interdisciplinary approach blending climatology, ecology, and public health is essential to evaluate and adapt control measures effectively under changing climate conditions.

### Modelling for decision making in schistosomiasis

Current models [85, 86] for schistosomiasis elimination have gaps in understanding transmission dynamics, infection heterogeneity, and the impact of climate change. Models should incorporate WASH interventions, assess drug resistance risks and treatment efficacy, and evaluate the disease's socio-economic effects. Effective elimination programs rely on health system capacity, vector control, co-infection management, and robust surveillance systems for outbreak detection and response. To optimize strategies, models must simulate surveillance and response while integrating environmental, biological, socio-economic, and health system factors. Addressing these modelling gaps is critical for informing comprehensive approaches to schistosomiasis elimination.

### Ensuring sufficient resources for schistosomiasis R&D

Investment in research and development is essential to advance understanding, develop innovative strategies, and address challenges like the threat of drug resistance in schistosomiasis control. Advocacy with policymakers, donors, and the private sector, alongside demonstrating the socioeconomic benefits of elimination, can drive critical support. Cross-sectoral governance and sustainable funding are vital, requiring collaboration across WASH, agriculture, environmental management, and animal health to address the parasite's lifecycle. Significant gaps remain in fostering collaboration, aligning priorities, and designing effective, localized interventions. Coordinated efforts in cross sectoral governance and resource mobilization will be crucial for achieving sustainable schistosomiasis elimination.

## Conclusions

The fight against schistosomiasis in the WHO Africa Region urgently requires addressing significant R&D gaps and operational challenges. Key knowledge gaps include the need for innovative diagnostics to replace low-sensitivity methods, better morbidity indicators, and new treatment strategies to improve access and address the threat of drug resistance. Operational research is vital to refine interventions in non-responsive areas, optimize WASH integration, and address behavioural and socio-economic barriers to treatment. Critical gaps also exist in understanding zoonotic transmission, schistosome species hybridization, and snail host ecology. Additionally, there is limited evidence on how climate change, environmental modifications, and cultural practices influence transmission in fast changing ecological settings. The lack of robust cross-sectoral governance frameworks and sustainable financing further hampers coordinated efforts across WASH, animal health, agriculture, and environmental management. A concerted effort by researchers, governments, funders, and international organizations is needed to prioritize and invest in these areas. Strengthened collaboration and resource allocation are essential to meet the 2030 NTD Roadmap targets, eliminate schistosomiasis as a public health threat, and achieve sustainable disease transmission interruption.

## Data Availability

Not applicable.

## Abbreviations

ARNTD: African Research Network for Neglected Tropical Diseases
eDNA: Environmental DNA
EPHP: Elimination of schistosomiasis as a public health problem
ESPEN: Expanded Special Project for Elimination of Neglected Tropical Diseases
GSA: Global Schistosomiasis Alliance
MDA: Mass Drug Administration
NNN: NTD NGO Network
NTD: Neglected tropical disease
PC: Preventive chemotherapy
R&D: Research and development
WASH: Water, sanitation, and hygiene
FGS: Female genital schistosomiasis
